# Flexible Electromagnetic Actuator with Liquid Metal Embedded in a Graphene Oxide/Thermoplastic Polyurethane Matrix

**DOI:** 10.3390/mi17080886

**Published:** 2026-07-25

**Authors:** Shufan Li, Yusuo Tian, Yang Zhang, Huatan Chen, Wenwang Li, Gaofeng Zheng, Xiang Wang

**Affiliations:** 1 School of Opto-Electronic and Communication Engineering, Xiamen University of Technology, Xiamen 361024, China; sfli@xmut.edu.cn; 2 School of Mechanical and Automotive Engineering, Xiamen University of Technology, Xiamen 361024, Chinaxmlww@xmut.edu.cn (W.L.); 3 Key Laboratory of Precision Actuation & Transmission (Fujian Province University), Xiamen 361024, China; 4 Xiamen Key Laboratory of Intelligent Manufacturing Equipment, Xiamen University of Technology, Xiamen 361024, China; 5 School of Intelligent Manufacturing (Pen-Tung Sah Institute of Micro-Nano Science and Technology), Xiamen University, Xiamen 361102, China; zheng_gf@xmu.edu.cn

**Keywords:** liquid metal, soft electromagnetic actuator, direct ink writing, electrospinning

## Abstract

Flexible electromagnetic actuators have attracted considerable attention for applications in soft robotics, adaptive manipulation, and human–machine interaction due to their fast response, large deformation capability, and inherent compliance. However, the concurrent application of high actuation performance and long-term cyclic durability remains a major challenge, particularly for liquid metal (LM)-based soft systems, where interfacial instability between LM conductors and polymer substrates often leads to performance degradation. In this work, we report a fabrication strategy in which patterned eutectic gallium–indium (EGaIn) liquid metal circuits are directly written onto electrospun graphene oxide/thermoplastic polyurethane (GO/TPU) nanofiber membranes. The incorporation of graphene oxide significantly enhances interfacial adhesion through hydrogen bonding interactions between oxygen-containing functional groups in GO and the native Ga_2_O_3_ layer on the LM surface, while the electrospun fibrous architecture further improves mechanical interlocking and structural stability. As a result, the fabricated actuator exhibits robust electromechanical performance, achieving a maximum bending deformation of 90° under a driving current of 0.8 A and maintaining stable operation over 2000 actuation cycles with negligible performance degradation. To further demonstrate its practical functionality, a soft robotic gripper was constructed based on the optimized actuator configuration. The gripper enables the stable grasping and lifting of objects with a weight up to seven times its own mass, while maintaining safe and compliant interaction with fragile objects. This work provides a simple yet effective strategy to simultaneously enhance actuation efficiency, interfacial stability, and mechanical reliability in LM-based GO/TPU flexible electromagnetic actuator systems, offering promising potential for next-generation soft robotic applications.

## 1. Introduction

Soft electromagnetic actuators (SEMA) have attracted extensive interest in soft robotics, marine exploration, biomedical devices, and human–machine interaction, owing to their fast dynamic response, wide operational bandwidth, and intrinsic compliance with biological tissues [1,2,3]. In particular, SEMAs incorporating liquid metal (LM) conductors have emerged as a promising class of soft transducers, as the high electrical conductivity and deformability of eutectic gallium–indium (EGaIn) enable robust current-carrying pathways while maintaining mechanical flexibility. When operated under external magnetic fields, these systems exhibit Lorentz-force-driven deformation, providing a compact and efficient route toward programmable soft actuation with enhanced spatial controllability and force outputs [4,5].

Despite these advantages, the practical implementation of LM-based soft actuators remains constrained by interfacial instability between liquid metal and flexible polymer substrates. The presence of a native Ga_2_O_3_ oxide layer on EGaIn, combined with its inherently high surface tension, leads to poor wettability and weak adhesion on conventional thermoplastic polyurethane (TPU) substrates. As a result, LM circuits are prone to delamination, interfacial slippage, and electrical failure under repeated mechanical deformation, significantly limiting device reliability and long-term operational stability [6,7]. Addressing this interfacial incompatibility is therefore critical for realizing durable and high-performance LM-based soft actuator systems.

To overcome this limitation, graphene oxide (GO) has been widely considered as an effective interfacial modifier due to its abundant oxygen-containing functional groups, including hydroxyl and carboxyl groups (i.e., -OH and -COOH), which can form strong hydrogen bonding interactions with the Ga_2_O_3_ layer on LM surfaces [8,9,10]. However, most previous strategies rely on surface coating or post-treatment methods, which provide limited interfacial integration and may not fully exploit the structural advantages of fibrous polymer networks.

In this work, we propose an integrated interfacial engineering strategy by incorporating GO directly into electrospun TPU nanofibers to construct a hierarchical GO/TPU substrate for LM-based soft actuators. The electrospun fibrous architecture not only increases surface roughness and effective contact area but also provides physical anchoring sites for liquid metal infiltration. Meanwhile, the chemically active GO domains enable strong interfacial interactions with the Ga_2_O_3_ oxide layer of EGaIn, resulting in significantly improved adhesion stability. The prepared GO/TPU nanofibrous substrate exhibits markedly reduced LM contact angle compared to pure TPU, and the patterned LM circuits remain firmly adhered even under full upside-down (180°) flipping, confirming robust interfacial integrity.

Based on this optimized substrate, flexible electromagnetic actuators were fabricated via direct ink writing of EGaIn circuits. The resulting devices demonstrate a maximum bending angle of 90° under a driving current of 0.8 A and exhibit stable electromechanical performance over 2000 cyclic loading-unloading cycles. Furthermore, structural and geometric optimization of actuator parameters further enhances thermal stability and actuation efficiency.

## 2. Materials and Methods

### 2.1. Preparation of GO/TPU Nanofibers

Graphene oxide (GO, Dazhan Nano (Guangdong) Co., Ltd., Zouping, China) was first dispersed in a mixed solvent system composed of N, N-dimethylformamide (DMF, Sinopharm Chemical Reagent Co., Ltd., Shanghai, China) and tetrahydrofuran (THF, Xilong Scientific, Shantou, China) at a volume ratio of 8:2. The mixture was ultrasonicated at 53 kHz for 1 h to ensure uniform GO exfoliation. Subsequently, thermoplastic polyurethane powder (TPU, 120 mesh, Zhongke New Material Plastic Chemical Co., Ltd., Luohe, China) was added to the GO dispersion to achieve a final solid content of 20 wt%. The mixture was magnetically stirred at 300 rpm for 8 h at room temperature, forming a homogeneous GO/TPU spinning solution without visible agglomerates.

Electrospinning was done by loading the GO/TPU solution into a 5 mL syringe mounted on a precision syringe pump. A positive voltage of +8 kV was applied to the needle, and a negative voltage of −2 kV was applied to a parallel-plate metal collector, creating a total potential difference of 10 kV. The needle-to-collector distance was fixed at 150 mm, and the solution flow rate was maintained at 12 μL/min. Under these conditions, continuous ultrafine fibers were deposited and stacked into a three-dimensional nanofibrous network. Highly oriented GO/TPU nanofiber membranes were collected as flexible substrates for subsequent actuator fabrication.

### 2.2. Direct Writing of Liquid Metal and Actuator Fabrication

**Figure 1:**
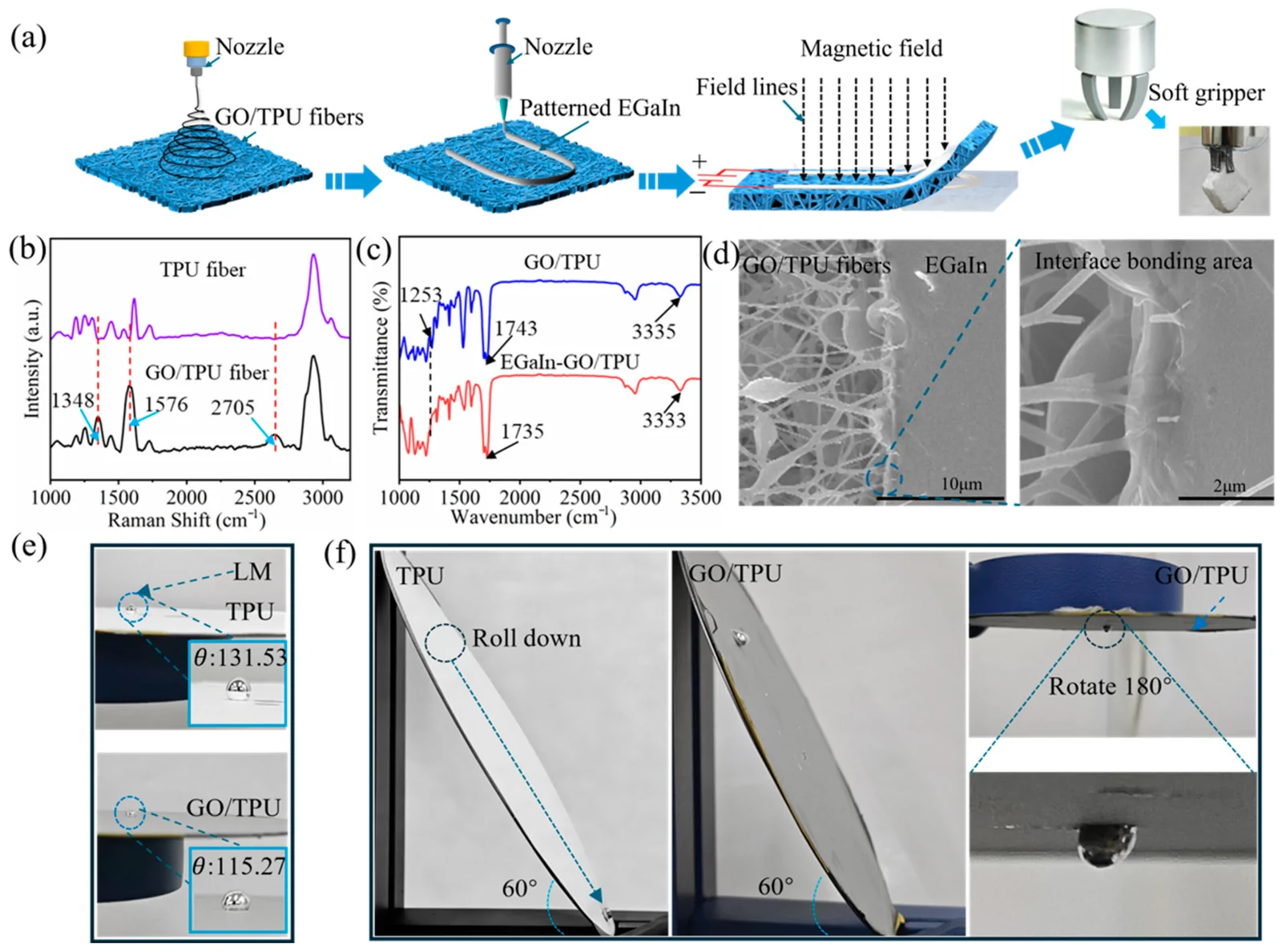
Fabrication and characterization of LM/GO-TPU composites. (**a**) Schematic diagram of flexible actuator fabrication. (**b**) Raman spectra of TPU and GO/TPU fibers, confirming successful incorporation of GO into the TPU matrix. (**c**) FTIR spectra of GO/TPU and EGaIn-GO/TPU, in which the disappearance of C-O peak (~1253 cm^−1^) confirms the existence of hydrogen bonding interactions between EGaIn-GO/TPU matrix. (**d**) Cross-sectional SEM topographical analysis of interfacial architectures. (**e**) Comparison of contact angles between TPU and GO/TPU. (**f**) Quantitative comparative evaluation of inclined-plane adhesion performance.

The overall fabrication process of the flexible electromagnetic actuator is schematically illustrated in Figure 1a. Eutectic gallium-indium alloy (EGaIn, 75 wt% Ga, 25 wt% In, Dongguan Dingguan Metal Technology Co., Ltd., Dongguan, China) was used as the liquid metal (LM) conductive material due to its excellent fluidity, high electrical conductivity, and low toxicity. The solution was loaded into a plastic syringe with a stainless-steel needle and extruded onto the electrospun GO/TPU membrane via direct ink writing (DIW). The high-precision motion platform guided the syringe along pre-designed circuit paths with positioning accuracy of ±0.3 μm.

A U-shaped LM pattern was designed as the basic actuation unit, with overall dimensions of 20 mm in length and 10 mm in width. The circuit consisted of two horizontal segments and two vertical segments, where only the horizontal segments contributed to the net Lorentz force under an external magnetic field due to current flow perpendicular to the magnetic field lines. To achieve stable and continuous LM lines, optimal DIW parameters were set at: liquid supply rate = 10 μL/min, platform moving speed = 3 mm/s, direct-writing height = 200 μm, and needle size = 19G. Uniform and stable LM circuits with an average line width of 207 ± 9.5 μm were successfully fabricated. It is worth noting that the 207 μm line width represents the finest continuous lines achievable under the current equipment configuration and processing conditions. We anticipate that even finer line widths could be realized through the adoption of advanced dispensing systems with higher pressure stability and optimization of ink rheology in future studies.

The GO/TPU-LM composite structure was then placed in an external magnetic field of magnetic flux density of approximately 0.3 T. When a driving current was applied to the LM circuit via a programmable linear DC power supply (DP832A, Rigol Technologies Inc., Suzhou, China), the Lorentz force bent the horizontal segments of the U-shaped circuit, thereby inducing controllable bending of the flexible actuator. The bending angle was defined as the angle between the tangent line at the actuator tip and the fixed base axis. By adjusting the current magnitude (0–0.8 A) and direction, bidirectional bending with angles exceeding 90° was achieved.

Three actuators are symmetrically mounted around a cylindrical magnet with a mutual angular separation of 120° to form a flexible three-legged gripper, enabling programmable grasping and releasing motions.

### 2.3. Characterization

The interfacial interactions between GO, TPU, and EGaIn were systematically characterized using multiple spectroscopic and microscopic techniques.

Raman spectra of pure TPU nanofibers, GO/TPU nanofibers, and EGaIn-GO/TPU composites (Figure 1b) were collected using a Raman spectrometer (DXR2xi, Thermo Fisher Scientific, USA) with a 532 nm excitation laser. The pure TPU fibers exhibited only characteristic organic polymer peaks (C–C, C=O, N–H), confirming the absence of carbonaceous species. In contrast, the GO/TPU nanofibers displayed distinct D (~1348 cm^−1^), G (~1576 cm^−1^), and 2D (~2705 cm^−1^) bands, confirming successful incorporation of GO into the TPU matrix. In addition, the intensity ratio of the D band to the G band (I_D_/I_G_) in GO/TPU spectra is used to quantitatively evaluate the defect density and oxidation degree of the material. The ratio is measured at 0.69, which was identical to that of pristine GO powder, indicating that the electrospinning process did not reduce or damage the oxygen-containing functional groups on GO. After incorporating EGaIn, the I_D_/I_G_ ratio increased to 0.98, suggesting partial reduction of oxygen-containing groups due to interactions with the Ga_2_O_3_ LM surface oxide layer.

Fourier transform infrared (FTIR) spectra were recorded in Figure 1c for GO/TPU and EGaIn-GO/TPU samples. The GO/TPU spectrum exhibited characteristic absorption bands corresponding to C–O stretching (~1253 cm^−1^), C=O stretching (~1743 cm^−1^), and N–H stretching (~3335 cm^−1^). After EGaIn deposition, the C–O stretching peak completely disappeared, while the C=O stretching peak downshifted from 1743 cm^−1^ to a lower wavenumber of 1735 cm^−1^. In contrast, the N–H stretching peak remained nearly unchanged, indicating that the amino groups were not involved in the interaction. The disappearance of the C–O peak and the shift in the C=O peak are typical spectroscopic signatures of hydrogen bond formation, confirming the existence of hydrogen bonding interactions between EGaIn and the GO/TPU nanofibers. While the FTIR spectroscopic evidence presented in this study is consistent with hydrogen bond formation between GO and the Ga_2_O_3_ layer, we acknowledge that direct techniques such as XPS or solid-state NMR would provide more definitive confirmation. This will be pursued in our future work.

The microstructure and interfacial architecture of the EGaIn-GO/TPU composite were examined using a field-emission scanning electron microscope (SEM, Sigma500, Carl Zeiss, Jena, Germany) at an accelerating voltage of 5 kV, as shown in Figure 1d. The SEM images show that the DIW EGaIn lines had an average width of 207 ± 9.5 μm. They exhibit characteristic micro-serrated edges that spatially couple with the randomly oriented distribution of GO/TPU nanofibers on the substrate surface. High-magnification cross-sectional SEM images reveal that the EGaIn traces were partially embedded within the nanofibrous network, while the GO fibers tightly wrapped around the EGaIn pathways. This distinctive embedded structure arises from the combined effects of physical confinement provided by the nanofibrous matrix and specific coordination interactions between the Ga_2_O_3_ layer on the GO/TPU surface and the oxygen-containing functional groups of GO. The resulting mechanically interlocked and chemically bonded interface imparts the LM circuit with excellent interfacial adhesion and stable electrical conductivity under repeated mechanical deformation.

Figure 1e shows the wettability of liquid metal on different nanofiber membranes, evaluated via the sessile drop method at ambient temperature and humidity. Five measurements were taken at randomly selected positions on each sample, and the average values were reported. On the pure TPU nanofiber membrane, the EGaIn droplet exhibited a nearly spherical shape with a high contact angle of 131.53° ± 2°, indicating poor wettability. In contrast, on the GO/TPU composite nanofiber membrane (0.5 wt% GO, disordered fiber configuration), the EGaIn droplet spread more readily and showed a significantly lower contact angle of 115.27° ± 1.7°. The improved wettability is attributed to the hydrogen bonding interactions between the oxygen-containing functional groups on GO and the Ga_2_O_3_ surface layer of EGaIn, which effectively reduce the solid–liquid interfacial energy.

Inclined-plane adhesion tests were performed by depositing EGaIn droplets onto horizontally positioned pure TPU and GO/TPU nanofibrous substrates, respectively, as shown in Figure 1f. The substrates were then gradually tilted until the droplets began to slide. On the pure TPU substrate, the EGaIn droplet maintained an approximately spherical shape with a relatively small contact area and completely slid off when the tilting angle reached 60°. In contrast, on the GO/TPU composite membrane, the EGaIn droplet exhibited a flattened semi-spherical morphology and remained firmly adhered even when the substrate was fully inverted (180° rotation), with no observable sliding or detachment. This remarkable adhesion strength confirms that the GO-modified nanofibrous substrate establishes a robust interfacial bonding with the EGaIn structure, primarily through hydrogen bonding and physical anchoring effects. The improved adhesion ensures that the LM circuit remains intact and functionally reliable under extreme deformation conditions, such as repeated large-angle bending and long-term cyclic actuation. While the inclined-plane critical sliding angle and cyclic endurance tests provide functionally relevant quantitative evidence of adhesion, we acknowledge that direct peel or shear tests would yield absolute adhesion strength values and will be pursued in future studies.

Taken together, these characterization results demonstrate that the introduction of GO into the TPU nanofibrous matrix significantly enhances both the wettability and adhesion strength toward liquid metal, providing a robust material foundation for the fabrication of high-performance and durable flexible electromagnetic actuators.

## 3. Results

### 3.1. Electromagnetic Actuation Performance and Simulation Validation

**Figure 2:**
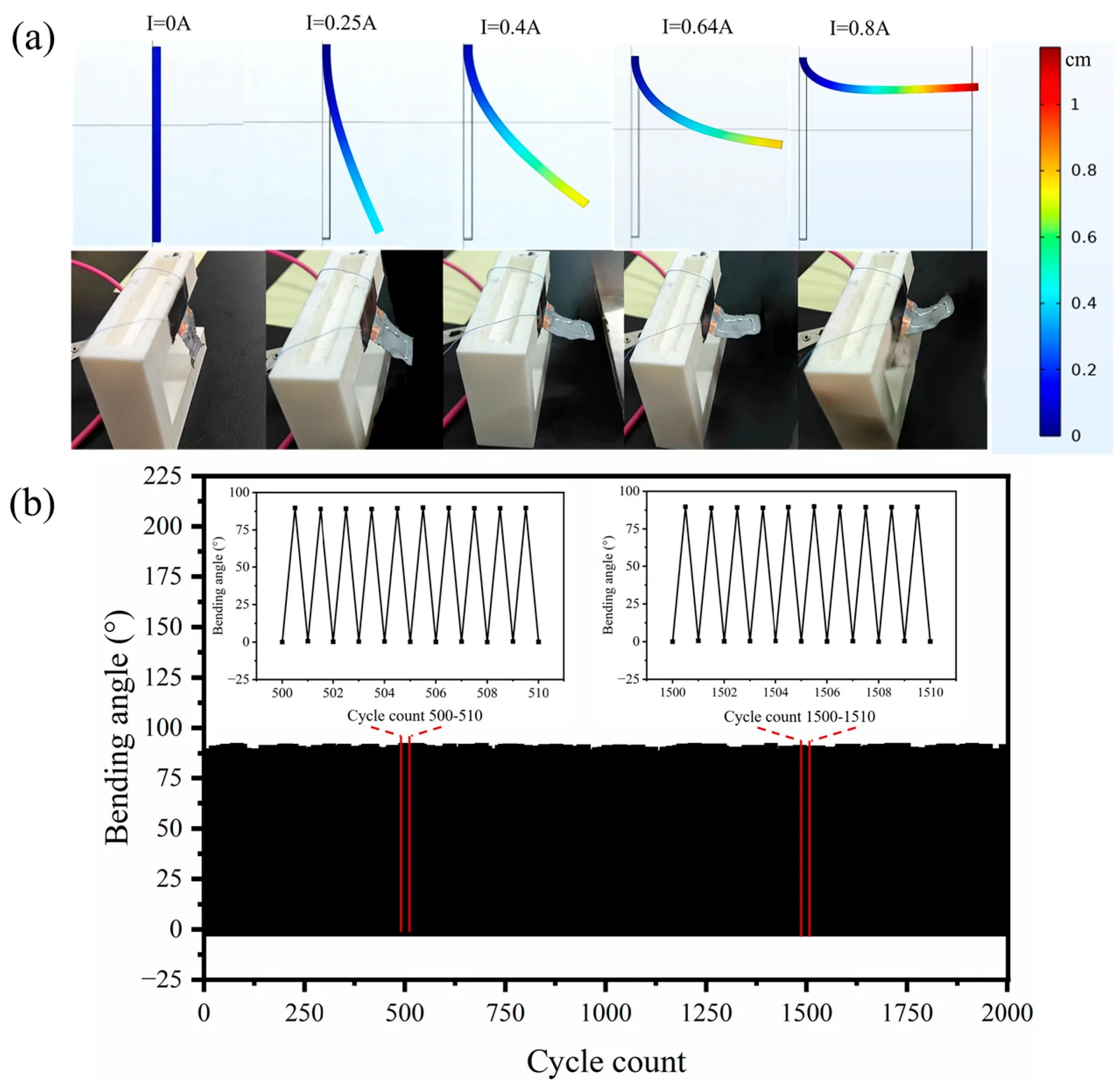
Fundamental actuation performance and cyclic stability of the flexible electromagnetic actuator. (**a**) Simulation and experimental comparison of bending angle as a function of driving current; (**b**) Cyclic electromechanical stability over 2000 on–off cycles (0 to 0.8 A), showing stable achievement of bending–straightening response with negligible performance degradation.

COMSOL 6.2 simulations were conducted to model the Lorentz-force-induced bending behavior of the liquid metal (LM) circuit on the GO/TPU nanofiber substrate. The finite element model incorporated a multi-physics coupling scheme that combined electromagnetic field simulation with solid mechanics. For the flexible substrate, the GO/TPU nanofiber membrane was assigned Young’s modulus E = 32 MPa and Poisson’s ratio ν = 0.48. The Lorentz force field generated within the LM circuit under specific current and external magnetic field conditions was accurately calculated via electromagnetic field simulation. Subsequently, this Lorentz force field was applied as a body load in the structural mechanics solver, and the static deformation and bending response of the actuator structure were solved through finite element analysis to predict its deformation configuration and bending angle. To evaluate the fundamental actuation performance and long-term reliability of the fabricated actuator, we first characterized its bending behavior and cyclic stability, as summarized in Figure 2.

The LM-patterned GO/TPU soft robotic actuator was assembled on a permanent magnet to construct the soft electromagnetic actuator (SEMA) device. Upon current application, the Lorentz force generated within the horizontal LM segment drove bending deformation of the composite actuator. The simulated deformation profiles agree well with experimental observations, and a bending angle of 90° was achieved at a driving current of 0.8A (Figure 2a). To quantitatively investigate the relationship between current input and bending deformation, multiple representative driving current values were selected for comparative analysis. At 0 A, the actuator remained straight without any observable bending. Within the current range of 0.25 A to 0.64 A, the bending degree progressively increased with increasing current. When the current reached the maximum value of 0.8 A, the actuator achieved a 90° bending angle. The measured bending state showed excellent consistency with the simulation results in terms of both deformation configuration and bending angle magnitude, consistent with the fundamental law of Lorentz force action. This strong agreement fully verifies the accuracy of the finite element model for predicting the deformation behavior of this type of electrically actuated soft actuator.

Control experiments were performed by reversing the current direction and flipping the magnetic field polarity; in each case, the bending direction reversed accordingly, confirming Lorentz-force-driven actuation. These observations, as shown in Table 1, are consistent with the directional response described above, though the corresponding photographic images are not shown due to redundancy in visual appearance.

### 3.2. Cyclic Electromechanical Durability and Stability

Actuation repeatability is a critical parameter for evaluating the operational reliability of flexible actuators. Owing to the nonlinear deformation characteristics of soft materials, repeatable actuation performance remains challenging. Therefore, cyclic actuation tests were conducted to assess the repeatability and stability of the proposed actuator.

To evaluate the electromechanical durability of the flexible electromagnetic actuator, rigorous cyclic fatigue tests were conducted under repeated current loading from 0 to 0.8 A. The actuator underwent 2000 continuous bending–recovery cycles between 0° and 90°. As presented in Figure 2b, the maximum bending angle remained within 90° ± 2° during cyclic actuation. After 2000 cycles, the actuator still maintained a stable maximum bending angle of 90° ± 5°, corresponding to an attenuation of less than 5%, indicating excellent actuation repeatability and cyclic stability.

No circuit fracture, liquid metal delamination, or interfacial failure was observed after cycling, and no noticeable fatigue cracks appeared on the substrate surface. The outstanding structural stability is attributed to the strong interfacial interaction between the LM circuit and the GO/TPU nanofiber substrate, arising from hydrogen bonding between the oxygen-containing functional groups of GO and the Ga_2_O_3_ oxide layer of EGaIn, together with the physical anchoring effect of the nanofibrous network. These results demonstrate the excellent mechanical robustness and long-term operational reliability of the actuator under repeated large-deformation conditions.

### 3.3. Optimization of Structural Parameters for Enhanced Actuation

**Figure 3:**
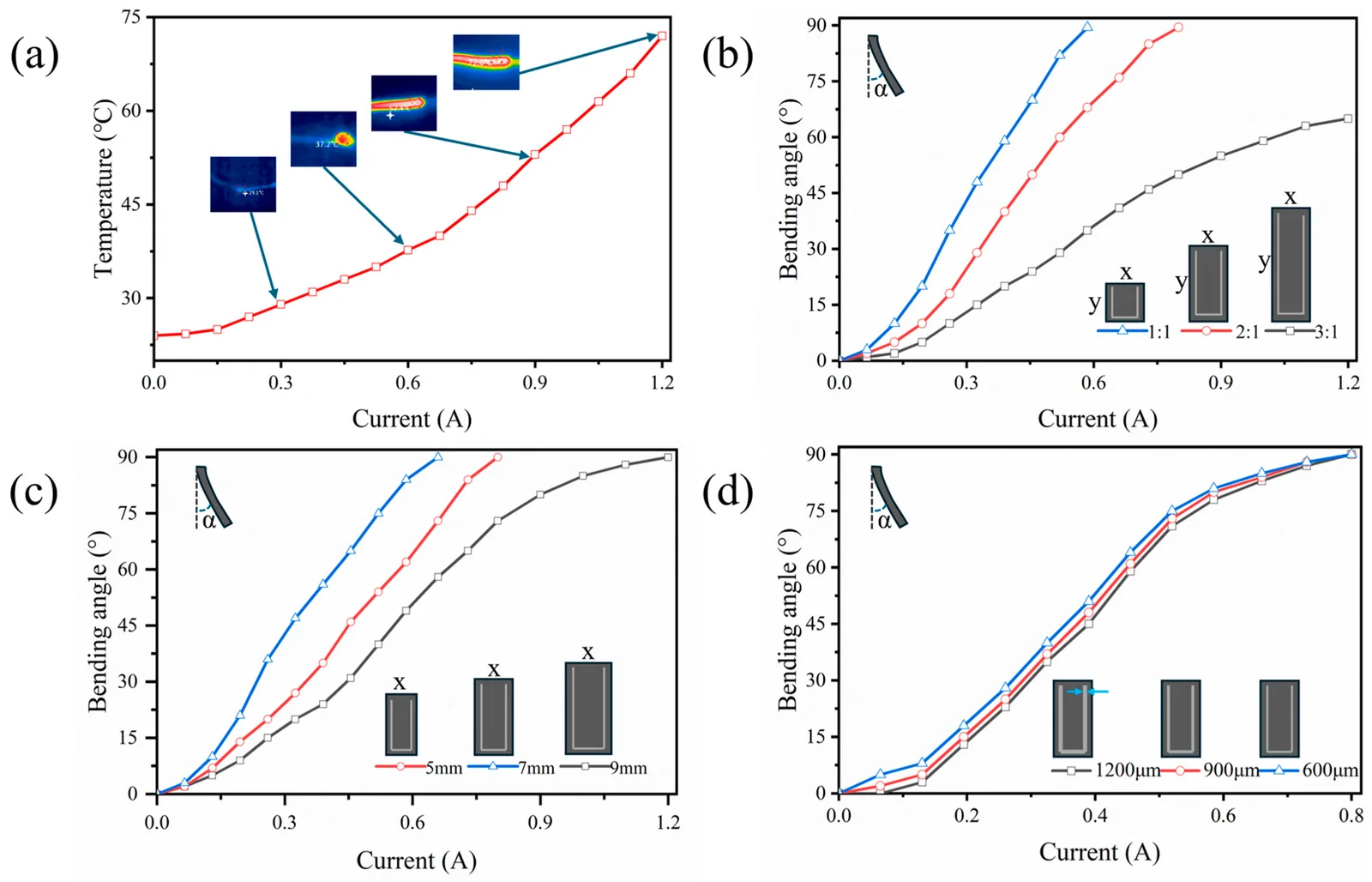
Systematic parametric optimization of the flexible electromagnetic actuator: (**a**) Temperature variation in the optimized GO/TPU-based actuator (thickness = 510 µm) as a function of driving current; (**b**) Effect of substrate aspect ratio on actuation bending performance. (**c**) Effect of actuator width. (**d**) Effect of printed LM circuit line width (1200 µm, 900 µm, and 600 µm respectively), represented by the blue arrows.

Having established the basic actuation capability and stability of the device, we next conducted a systematic parametric optimization to identify the optimal geometric configuration that maximizes actuation efficiency, as shown in Figure 3. To achieve miniaturized yet high-performance actuation, the actuator geometry was optimized by balancing structural stability, thermal tolerance, and electromagnetic deformation capability. The geometric parameters of the flexible actuator investigated in this study include the GO/TPU substrate thickness, the aspect ratio of the substrate dimensions (y:x, where y and x denote the length and width, respectively), the substrate width (x), and the LM circuit line width (w).

Substrate thickness plays a crucial role in determining the thermal–mechanical stability of the actuator under current loading. Figure 3a presents the temperature variation as a function of driving current for the 510 µm-thick actuator, confirming its stable thermal response under electrical actuation. The 510 µm actuator achieves an optimal balance between thermal stability and actuation capability, maintaining structural integrity without thermally induced deformation even at a driving current of 1.2 A while preserving excellent electromagnetic bending performance. Accordingly, the electrospun GO/TPU actuator with a thickness of 510 µm was selected as the optimal configuration for subsequent experiments. Supplementary Material (Figure S1) further confirms that non-optimized substrate thickness significantly degrades actuator performance, where both insufficiently thin and excessively thick substrates lead to thermal instability or reduced actuation efficiency, respectively.

From the perspective of practical application safety, the thermal behavior of the actuator was further evaluated. The actuator exhibits a temperature rise of approximately 5 °C at 0.8 A during normal operation. However, at higher currents or under extended operation, the surface temperature may exceed 50 °C, making it unsuitable for direct and prolonged skin contact in human–machine interaction scenarios at this stage. For applications requiring human proximity, current-limiting circuits or thermally insulating layers are recommended to mitigate heat accumulation.

The aspect ratio (y:x) of the actuator significantly influences its bending behavior, as shown in Figure 3b. Based on the optimized substrate thickness of 510 µm, actuators with aspect ratios of 1:1, 2:1, and 3:1 were fabricated and evaluated under a pre-established magnetic field. The 1:1 configuration exhibited limited deformation due to insufficient effective lever length, resulting in the smallest bending angle (<60° at 0.8 A). In contrast, the 3:1 configuration provided enhanced electromagnetic response and achieved a 90° bending angle at 0.6 A; however, it suffered from reduced structural stability and a tendency toward buckling under actuation. The 2:1 configuration offered the best balance between deformation capability and mechanical stability, enabling stable 90° bending without structural collapse. Therefore, an aspect ratio of 2:1 was selected as optimal.

With the thickness (510 µm) and aspect ratio (2:1) determined, the effect of actuator width (x) was further investigated (Figure 3c). Actuators with widths of 5 mm, 7 mm, and 9 mm (corresponding to lengths of 10, 14, and 18 mm, respectively) were evaluated. Among them, the 7 mm sample exhibited the most efficient force-deformation response, achieving 90° bending at 0.8 A, outperforming both narrower and wider configurations. Comparative current-angle analysis further confirmed 7 mm as the optimal width for maximizing actuation performance within the established geometric framework.

Finally, under the optimized structural parameters (h = 510 µm, y:x = 2:1, x = 7 mm), the influence of LM circuit line width (w) was examined (Figure 3d). The 207-μm lines fabricated in Section 2.2 demonstrate the capability of our DIW process to produce continuous features well below the sub-millimeter scale. As such, the same printing approach is fully adequate for constructing the practical device structures with line widths in the sub-millimeter range (0.6–1.2 mm), which were designed to meet the current-carrying requirements for actuation. Accordingly, line widths of 0.6 mm, 0.9 mm, and 1.2 mm were tested. The results indicate that the line width has negligible impact on the fundamental bending performance within this range. However, considering device reliability, wider channels increase the risk of liquid metal leakage during operation. Therefore, a line width of 0.6 mm was selected as the optimal design.

Overall, the optimized configuration of the flexible electromagnetic actuator was determined as h = 510 µm, y:x = 2:1, x = 7 mm, and w = 0.6 mm, providing a balanced combination of mechanical stability, actuation efficiency, and operational reliability.

### 3.4. Functional Demonstration: Flexible Gripper Application

To demonstrate the practical functionality of the optimized flexible electromagnetic actuator, a compliant soft electromagnetic gripper was constructed by symmetrically assembling three actuators at 120° intervals on a cylindrical permanent magnet (20 mm diameter, 20 mm height). This radial configuration enables coordinated actuation through the superposition of Lorentz forces generated by each individual actuator, resulting in a collective centripetal motion suitable for adaptive grasping. The proposed gripper adopts a three-limb architecture, where each limb functions as an independently addressable soft actuator capable of programmable bending deformation via controlled current input.

**Figure 4:**
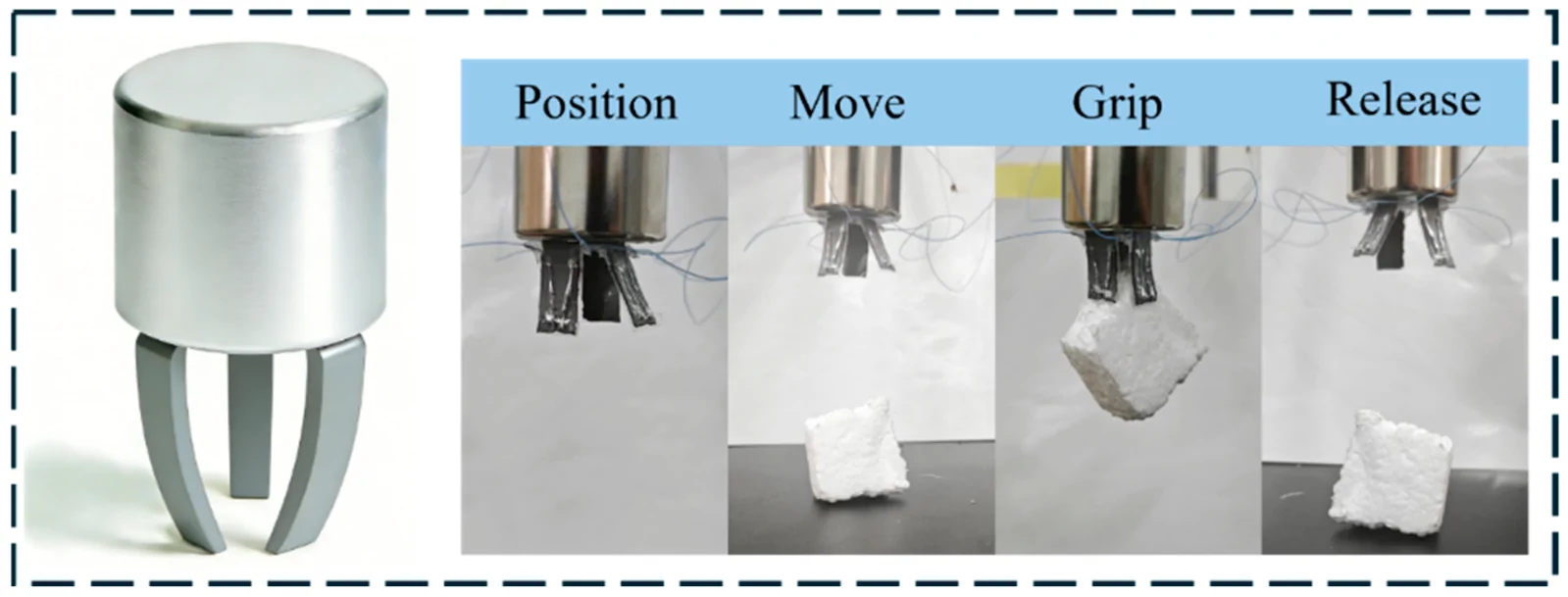
Grasping process of the flexible three-actuator gripper.

As shown in Figure 4, coordinated current regulation enables tunable and repeatable grasping behavior. The gripper exhibits stable operation and effective load handling under static testing conditions using foam blocks as representative soft targets. At a driving current of 0.8 A, the gripper was able to reliably grasp and lift loads exceeding five times its own mass, demonstrating strong force output while maintaining structural compliance. Following further optimization of the liquid metal circuit design (*n* = 2 double-row U-shaped configuration), as shown in Supplementary Material Figure S2, the load-bearing capacity was further enhanced, allowing stable operation under loads exceeding seven times its self-weight.

The improved performance is attributed to the optimized current distribution and enhanced electromagnetic force generation enabled by the refined circuit architecture, which improves force uniformity across the three actuating arms. This demonstrates the critical role of the LM circuit design in determining the overall mechanical output and functional efficiency of the system.

Importantly, the gripper maintains excellent structural integrity during repeated grasping and release cycles. The compliant nature of the actuator ensures uniform stress distribution at the contact interface, effectively minimizing localized stress concentration and preventing damage to delicate objects. No observable damage or deformation was detected in the foam targets throughout the manipulation process, confirming the suitability of the device for gentle yet high-force grasping tasks.

Overall, the proposed soft gripper integrates high load capacity with safe manipulation capability, achieving both mechanical robustness and compliance. These results highlight the potential of the developed flexible electromagnetic actuator for soft robotic applications, particularly in adaptive manipulation, human–robot interaction, and the handling of fragile or irregular objects.

## 4. Discussion

This work presents a flexible electromagnetic actuator system based on GO/TPU electrospun nanofibrous substrates integrated with patterned EGaIn liquid metal circuits. The introduction of GO into the TPU fibrous network significantly enhances interfacial interaction with the liquid metal, improving adhesion stability and structural robustness. This is primarily attributed to hydrogen bonding between oxygen-containing functional groups in GO and the native Ga_2_O_3_ oxide layer on the EGaIn surface, together with mechanical interlocking provided by the nanofibrous architecture.

Based on this material platform, a Lorentz-force-driven soft actuator was developed and systematically optimized in terms of structural thickness, aspect ratio, actuator width, and liquid metal circuit line width. The optimized actuator achieved a maximum bending angle of 90° at a driving current of 0.8 A, while maintaining stable and repeatable performance over 2000 actuation cycles, demonstrating excellent electromechanical reliability and fatigue resistance. Thermal-mechanical analysis further confirmed that appropriate structural design effectively suppresses thermally induced deformation while preserving efficient electromagnetic actuation.

In addition, the functional capability of the actuator was extended to a multi-unit soft robotic system. By integrating three actuators at 120° intervals on a cylindrical permanent magnet, a compliant gripper was constructed, enabling coordinated centripetal motion through Lorentz-force coupling. The gripper demonstrated stable grasping and release of objects exceeding five times its own weight, and up to seven times after circuit optimization, while maintaining safe manipulation of fragile objects without causing structural damage.

Overall, this study provides a simple yet effective design strategy for high-performance LM-GO/TPU-based soft actuators. The combination of interfacial engineering, electromagnetic actuation, and structural optimization offers a promising route toward robust, scalable, and multifunctional soft robotic systems for applications in adaptive manipulation, human–machine interaction, and flexible automation. Thermal management considerations, as discussed in Section 3.3, define the operational boundaries of the actuator for human-centric applications, and the proposed mitigation strategies provide a pathway toward safer device integration, particularly for scenarios involving direct human proximity or extended operation.

## Figures and Tables

**Table 1:** Control experiments verifying Lorentz-force-driven actuation via current and magnetic field direction reversal.

Condition	Current Direction	Magnetic Field Direction	Observed Bending Direction	Bending Angle
Case 1	Forward	Upward	Bends to sideA	~90° (at 0.8 A)
Case 2	Reverse	Upward	Bends to sideB	~90° (at 0.8 A)
Case 3	Forward	Downward	Bends to sideB	~90° (at 0.8 A)
Case 4	Reverse	Downward	Bends to sideA	~90° (at 0.8 A)

where sideA is denoted as the right side of the samples in Figure 2a, sideB denotes the opposite side after physically flipping the sample.

## Data Availability

Data will be made available on request.

## Supplementary Materials

The following supporting information can be downloaded at: https://www.mdpi.com/article/10.3390/mi17080886/s1, Figure S1: Thermal response of GO/TPU substrates with non-optimized thicknesses under current loading; Figure S2: Performance evaluation of the flexible gripper.
