# Supplementary material for: Flexible Electromagnetic Actuator with Liquid Metal Embedded in a Graphene Oxide/Thermoplastic Polyurethane Matrix
Source: Micromachines (Basel). 2026 Jul 25;17(8):886. doi: 10.3390/mi17080886 (PMC13515088; doi:10.3390/mi17080886)
Supplement: Supplementary file 1 [file micromachines-17-00886-s001.zip › micromachines-4406719-supplementary.pdf]

Supplementary Information.

Flexible Electromagnetic Actuator with Liquid Metal Embedded in a Graphene Oxide/Thermoplastic Polyurethane Matrix

Shufan Li [1], Yusuo Tian [2], Yang Zhang [2], Huatan Chen [2], Wenwang Li [2,3,4], Gaofeng Zheng [5] and Xiang Wang [2,3,4,*]

[1] School of Opto-Electronic and Communication Engineering, Xiamen University of Technology, Xiamen 361024, China ; sfli@xmut.edu.cn

[2] School of Mechanical and Automotive Engineering, Xiamen University of Technology, Xiamen 361024, China; xmlww@xmut.edu.cn (W.L.)

[3] Key Laboratory of Precision Actuation & Transmission (Fujian Province University), Xiamen 361024, China

[4] Xiamen Key Laboratory of Intelligent Manufacturing Equipment, Xiamen University of Technology, Xiamen 361024, China

[5] School of Intelligent Manufacturing (Pen-Tung Sah Institute of Micro-Nano Science and Technology), Xiamen University, Xiamen 361102, China ; zheng_gf@xmu.edu.cn

[*] Correspondence: wx@xmut.edu.cn

Contents:

Figure S1. Thermal response of GO/TPU substrates with non-optimized thicknesses under current loading

Figure S2. Performance evaluation of the flexible gripper.

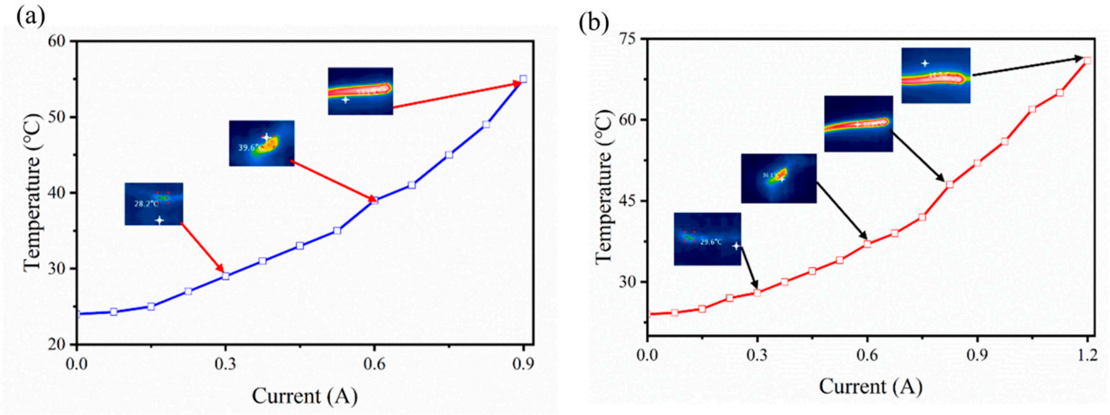

Figure S1. Thermal response of GO/TPU substrates with non-optimized thicknesses under current loading: (a) 320 μm substrate showing thermal instability and deformation under electrical driving; (b) 725 μm substrate showing reduced actuation efficiency due to increased stiffness.

The experimental results reveal that non-optimized substrate thicknesses substantially degrade actuator performance. The 320 μm substrate, characterized by insufficient structural rigidity, undergoes thermal damage and uncontrolled deformation when subjected to a continuous 0.9 A current. In contrast, the 725 μm substrate exhibits compromised bending capability and diminished energy conversion efficiency owing to excessive stiffness.

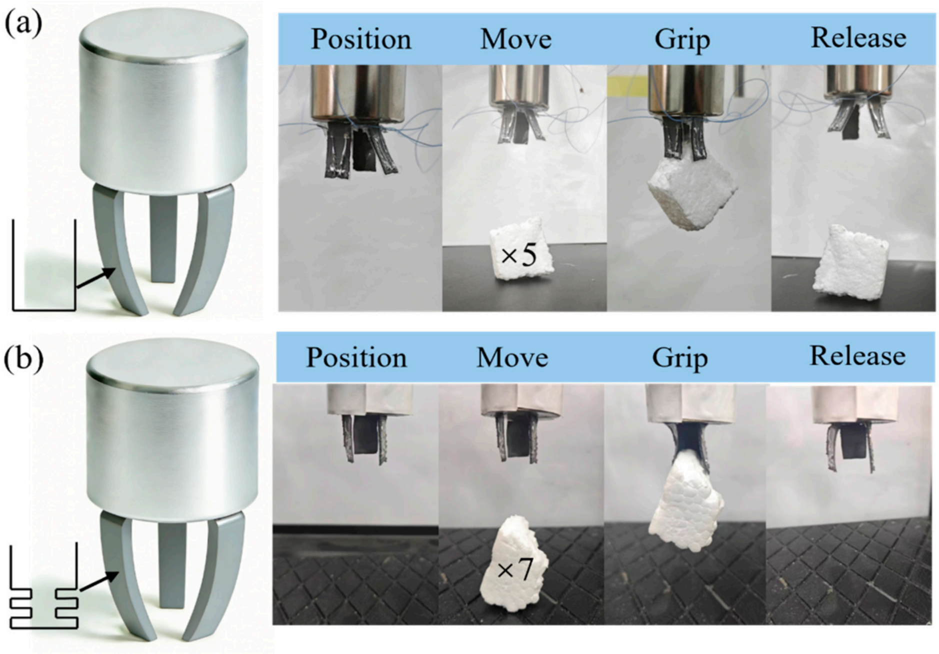

Figure S2. Performance evaluation of the flexible gripper: (a) U-shaped liquid metal (LM) actuator; (b) $n = 2$ double-row U-shaped LM actuator.

The fabricated gripper adopts a symmetric three-leg architecture, in which three flexible actuators are assembled in a radial configuration at 120° intervals. Systematic static loading tests were conducted using lightweight foam blocks to evaluate the operational performance of the gripper. The device can safely manipulate foam objects while maintaining structural integrity under loading conditions, effectively mitigating stress concentration due to its compliant design.

At a driving current of 0.8 A, the U-shaped LM circuit gripper remains stable even when the applied load exceeds 5 times its own weight. After circuit optimization (n = 2 double-row U-shaped configuration), the load-bearing capacity is further improved, enabling stable operation under loads exceeding 7 times its own weight. These results demonstrate the critical role of LM circuit design in enhancing actuation performance and highlight the feasibility and potential of force-coordinated soft robotic design strategies.
